# 82例伴Coombs试验阳性血细胞减少患者临床特征及预后分析

**DOI:** 10.3760/cma.j.issn.0253-2727.2021.06.015

**Published:** 2021-06

**Authors:** 璇 郑, 英起 邵, 星鑫 李, 金波 黄, 美丽 葛, 敏 王, 朋 金, 能 聂, 静 张, 以州 郑

**Affiliations:** 中国医学科学院、北京协和医学院血液病医院（中国医学科学院血液学研究所），实验血液学国家重点实验室，国家血液系统疾病临床医学研究中心，天津 300020 State Key Laboratory of Experimental Hematology, National Clinical Research Center for Blood Diseases, Institute of Hematology & Blood Diseases Hospital, Chinese Academy of Medical Sciences & Peking Union Medical College, Tianjin 300020, China

血细胞减少病因复杂多样，确诊较困难，属排除性诊断。临床上此类患者常见一项或多项血细胞减少且合并Coombs试验阳性，但网织红细胞比例（Ret％）正常或稍高。此部分患者体液免疫异常，其临床意义未明，预后尚未见报道。故笔者回顾性分析82例伴Coombs试验阳性血细胞减少患者的病例资料，总结其临床特征及转归，现报道如下。

## 病例与方法

一、研究对象

2009年7月1日至2020年6年30日于我科住院伴Coombs试验阳性的血细胞减少患者，其诊断及疗效判断标准参照《血液病诊断及疗效标准》第4版[1]。风湿疾病诊断符合中华医学会风湿病学分会相应的诊断标准并结合风湿科医师会诊意见。排除标准：自身免疫性溶血性贫血、纯红细胞再生障碍性贫血、原发免疫性血小板减少性、Evans综合征、淋巴增殖性肿瘤、原发性骨髓纤维化、非血液系统肿瘤及肝硬化等继发性血细胞减少患者。

二、辅助检查

1. 常规检查：血常规+网织红细胞绝对计数（Ret）、生化、贫血四项及腹部B超。

2. 溶血检查：血浆游离血红蛋白、血浆结合珠蛋白检测，直接Coombs试验，冷凝集素试验，酸化甘油溶血试验，红细胞盐水渗透脆性试验及阵发性睡眠性血红蛋白尿症（PNH）克隆检测。

3. 免疫学检查：淋巴细胞亚群、免疫球蛋白定量分析（IgG、IgA、IgM）、补体C3和C4、类风湿因子、抗链球菌溶血素O、抗核抗体（ANA）滴度及可提取性核抗原（ENA）抗体谱（nRNP/Sm、dsDNA、Sm、SS-A、SS-B、SCL-70、Jo-1、His、Ro-52、着丝点、核小体、核糖体P蛋白抗体）。

4. 骨髓检查：细胞形态学、活检、免疫分型和染色体核型分析。

三、治疗方案

诊断获得性骨髓衰竭患者均予环孢素A联合左旋咪唑、达那唑/司坦唑醇等免疫抑制及促造血治疗，并补充造血原料。继发组患者与风湿免疫性疾病相关，转诊至风湿免疫科并随访。

四、统计学处理

应用SPSS 26.0进行统计学分析。随访终点为移植、死亡或2020年10月30日。患者临床特征中符合正态分布的连续性变量采用均数±标准差描述，组间差异比较采用*t*检验；非正态分布、方差不齐的连续变量以中位数（范围）表示，差异比较采用非参数Mann-Whitney *U*检验；计数资料采用百分比描述，组间比较采用*c*_2_检验或Fisher精确概率法；总体生存率采用Kaplan-Meier法估算，组间比较用Log-rank检验。*P*<0.05为差异有统计学意义。

## 结果

一、病例资料

共82例伴Coombs试验阳性的血细胞减少患者符合纳入标准，其中男性38例，女性44例，中位年龄42.5（8～71）岁。获得性骨髓衰竭患者（BMF组）58例，其中再生障碍性贫血（AA）17例，低中危骨髓增生异常综合征（MDS）31例和PNH 10例。继发性血细胞减少患者（继发组）24例，其中未分化结缔组织病10例，系统性红斑狼疮（SLE）4例，类风湿性关节炎5例，白塞病2例，干燥综合征1例，结节性红斑1例，抗磷脂综合征1例。

二、两组临床特征分析

1. 一般特征比较：继发组女性比例明显高于BMF组（83％对41％，*P*＝0.001）。该组患者以轻中度贫血［HGB中位数78（31～129）g/L］为主，其WBC水平略低于正常，而PLT中位数明显高于BMF组［71（4～384）×10^9^/L对31.5（4～401）×10^9^/L，*P*＝0.020］。继发组脾脏肿大更为常见，其阳性率高于BMF组（38％对7％，*P*＝0.002）（表1）。

**表1:** 伴Coombs试验阳性血细胞减少症患者临床特征

临床特征	BMF组（58例）	继发组（24例）	*P*值
性别［男（％）］	34（59）	4（17）	0.001
年龄［岁，*M*（范围）］	42（8～71）	44（11～70）	0.834
WBC［×10^9^/L，*M*（范围）］	2.31（0.23～9.85）	3.15（0.77～11.92）	0.082
HGB［g/L，*M*（范围）］	70（27～141）	78（31～129）	0.215
PLT［×10^9^/L，*M*（范围）］	31.5（4～401）	71（4～384）	0.020
Ret［×10^9^/L，*M*（范围）］	37.1（1.3～300.8）	53.6（2.0～305.4）	0.285
肝大［例（％）］	10（17）	1（4）	0.221
脾大［例（％）］	4（7）	9（38）	0.002

注：BMF组：获得性骨髓衰竭患者；继发组：继发性血细胞减少患者；Ret：网织红细胞绝对计数

2. 免疫学检查比较：BMF组患者淋巴细胞比例显著高于继发组患者［58.3％（16.4％～94.5％）对34.6％（5.4％～73.7％），*P*<0.001］，而CD19^+^ B细胞比例（9.3％）却明显低于继发组患者［9.3％（0.3％～27.1％）对14.0％（1.2％～35.6％），*P*＝0.010］。继发组患者风湿免疫相关的指标，如ANA滴度/阳性率、ENA抗体谱阳性率及IgG水平明显高于BMF组患者，但继发组补体C3、C4水平低于BMF组（*P*值均<0.05）（表2）。

**表2:** 伴Coombs试验阳性血细胞减少症患者免疫学检查

免疫学检查	BMF组（58例）	继发组（24例）	*P*值
淋巴细胞比例［％，*M*（范围）］	58.3（16.4～94.5）	34.6（5.4～73.7）	<0.001
CD19^+^ B细胞比例［％，*M*（范围）］	9.3（0.3～27.1）	14.0（1.2～35.6）	0.014
ANA滴度［*M*（范围）］	90（0～3200）	1000（0～10000）	<0.001
ANA阳性［例（％）］	30（52）	21（88）	0.002
ENA抗体谱阳性［例（％）］	16（28）	17（71）	0.001
补体C3（g/L，*x*±*s*）	1.1±0.3	0.6±0.3	0.001
补体C4（g/L，*x*±*s*）	0.26±0.10	0.13±0.06	0.001
IgG［g/L，*M*（范围）］	11.05（1.69～25.30）	18.35（3.89～82.20）	0.005

注：BMF组：获得性骨髓衰竭患者；继发组：继发性血细胞减少患者；ANA：抗核抗体；ENA：可提取性核抗原

3. BMF组临床特征：本组患者共14例合并肝脾肿大，且多为轻度肿大。其中1例PNH患者脾重度大（肋缘下10 cm），入院前2周出现发热伴黄疸，高度怀疑合并脾栓塞和（或）脾淤血。另外，仅1例难治性贫血伴多系病态造血（MDS-RCMD）患者肝、脾均肿大。48例（83％）患者既往有输血史，12例（21％）患者入院前2周有发热病史并予青霉素类或头孢菌素类等抗生素治疗，4例（7％）患者有丙种球蛋白治疗史（3个月内）。

三、血液学反应及预后

共有17例BMF患者复查Coombs试验，11例（65％）转阴。余6例阳性患者中，1例效价较前升高，1例持平，4例较前降低。Coombs试验效价转阴或降低的15例患者，其中10例（67％）经免疫抑制及促造血治疗后脱离输血；另5例均为MDS及PNH患者，尚未获得血液学缓解，仍依赖输血。

共随访49例患者，其中39例规范服药。截至末次随访，总有效率为79.5％（31/39），5例死亡；8例未规律服药患者有4例死亡，另4例患者带病生存，且依赖血制品输注。本组患者5年总体生存率为（79.8±7.3）％，服药患者生存率与未服药组差异无统计学意义（*P*＝0.090）。另2例患者行骨髓移植治疗获得血液学缓解。

## 讨论

临床上血细胞减少患者极常见，其诊断需多学科鉴别。近来我们发现Coombs试验阳性但Ret比例正常或稍高的血细胞减少患者体液免疫指标异常，往往对诊断造成一定干扰。本文首次就其临床表现、疗效及长期生存进行总结，以期更深入了解此组疾病。

据Serris等[2]报道，系统性红斑狼疮（SLE）合并血细胞减少患者HGB中位数为85（69～98）g/L，国内严煜林及周茂松[3]关于SLE合并全血细胞减少患者的研究提示：此类患者血象特征为中度贫血、轻度白细胞、血小板降低［HGB 74±14 g/L、WBC（3.3±0.7）×10^9^/L及PLT（60±29）×10^9^/L］，本研究结果与上述报道相符，此组患者骨髓增生程度及细胞形态均大致正常，偶见红系形态异常，由此可除外BMF等疾病，加之其ANA滴度/阳性率和ENA抗体谱阳性率较高，不难诊断为继发性血细胞减少。

虽然BMF组患者Coombs试验阳性，但其中位总胆红素、间接胆红素、乳酸脱氢酶以及Ret水平均在正常范围内，无红细胞破坏的间接证据。此特征不同于少数个案报道中MDS合并自身免疫性溶血性贫血的情况[4]–[5]，后者除Coombs试验阳性外，常合并高胆红素血症，Ret明显升高及脾脏肿大。本组患者ANA效价多为1∶100～1∶320（参考范围0～1∶100），风湿免疫病证据不足，结合患者骨髓相关特征，仍考虑BMF。有文献指出ANA阳性常见于自身免疫性疾病，亦可见于感染、肿瘤患者及正常人群[6]。1995年，国外学者Enright等[7]对221例MDS患者进行回顾性分析发现，40例（其中30例合并自身免疫性疾病）出现免疫学指标异常，以ANA最常见（35％），Coombs试验阳性比例达15％。两者虽无明显相关性，但Coombs试验及ANA均阳性提示是否存在体液免疫异常仍未确定。本研究BMF组ANA阳性患者占52％，亦提示两者间的潜在联系，但尚待进一步研究证实。

国外学者曾报道，健康人Coombs试验阳性率随着年龄增长而增加[8]。Coombs试验阳性率在正常人群中占1∶1000～1∶14 000，而在住院患者中可高达7％～8％[9]。本研究患者中位年龄42.5岁，亦提示其年龄相关性。如下因素可与Coombs试验阳性相关，除免疫系统调节紊乱产生自身抗体外，还包括：①输血因素[9]：受血者体内存在同种抗体与献血者红细胞膜抗原发生反应，或献血者血浆、红细胞制品等存在同种抗体与受血者红细胞膜抗原发生反应；②母亲血液内同种抗体经由胎盘附着于胎儿红细胞膜[10]；③高球蛋白血症[11]或输注丙种球蛋白治疗后等非特异性蛋白吸附于红细胞表面；④药物因素[12]，如青霉素、头孢菌素等抗菌药物能与免疫性蛋白吸附而结合红细胞膜上。本研究显示：83％的BMF组患者就诊时有输血史，21％的患者在入院前2周因发热行抗感染治疗，亦有7％的患者曾有丙种球蛋白治疗史，此为Coombs试验阳性的可能因素。经免疫抑制及促造血治疗6个月后，复查Coombs试验的BMF患者中，88％的患者其效价转阴或降低，亦能佐证上述推测。

有学者认为Coombs试验阳性与血清IgG水平呈正相关。B细胞异常可引起丙种球蛋白增多和器官特异性、非特异性自身抗体的分泌，如多发性骨髓瘤患者常出现Coombs试验阳性。该反应是由于单克隆免疫球蛋白被动吸附于红细胞表面，通常不会产生溶血[13]。继发组患者B细胞水平偏高亦提示其相关性。而BMF组患者外周血B淋巴细胞比例、IgG、补体C3和C4水平基本正常，所以体液免疫机制是否参与其中仍未可知。Coombs试验阳性可能由于全身性炎症导致非特异性抗体吸附于红细胞膜表面，却不与红细胞膜蛋白特异性结合（即它们不识别并结合特定的红细胞抗原），不会导致免疫球蛋白构象改变，亦没有补体参与，故并不加速红细胞破坏，通常不会引发溶血[10]，本组患者补体水平基本正常可证实这一判断。

随访49例BMF组患者，79.5％获得血液学缓解，与既往研究报道结果相近[14]–[15]，提示此类患者虽存在ANA及Coombs试验阳性等混杂因素，但适时给予免疫抑制及促造血治疗，仍可获得良好治疗反应，5年总体生存率达（79.8±7.3）％。服药及未服药患者生存率差异无统计学意义，可能与未服药组样本量较小有关，尚待进一步大宗病例长期随访研究。

综上，伴Coombs试验阳性血细胞减少患者，IgG水平和ANA滴度明显升高，补体C3、C4水平降低，常合并明显脾脏肿大，血细胞减少考虑为继发于风湿免疫疾病。伴Coombs试验阳性的骨髓衰竭性疾病，诊断须排除继发性因素，治疗上应积极给予经典的免疫抑制及促造血方案。Coombs试验阳性提示其可能存在体液免疫异常，具体发病机制有待进一步深入探讨。
